# The use of consumer-grade physical activity monitors: Insight into psychosocial determinants and technology acceptance

**DOI:** 10.1016/j.conctc.2025.101516

**Published:** 2025-06-30

**Authors:** Brenda A.J. Berendsen, Rianne H.J. Golsteijn, Lilian Lechner, Catherine Bolman, Denise A. Peels

**Affiliations:** a Department of Nutrition and Movement Sciences, Institute of Nutrition and Translational Research in Metabolism, Faculty of Health Medicine and Life Sciences, Maastricht University, PO Box 616, 6200 MD, Maastricht, the Netherlands; b Department of Health Psychology, Faculty of Psychology, Open University of the Netherlands, the Netherlands

**Keywords:** Physical activity, Wearables, Reach, Enrollment, Intervention research, Psychosocial determinants, Technology acceptance

## Abstract

**Background:**

Consumer-grade physical activity (PA) monitors are used to optimize enrollment in clinical trials, to evaluate PA behavior, or to promote PA. Insight is needed into characteristics of people who use PA monitors and who do not, to enhance recruitment and generalizability of trials. We assessed demographics, psychosocial determinants and technology acceptance of (non-)users of PA monitors.

**Methods:**

Dutch speaking adults were recruited via social media, email and personal contact. In an online questionnaire 533 participants (70 % women, age 43 ± 14 years) reported PA monitor use, PA and psychosocial determinants, and technology acceptance of PA monitors. Concepts were derived from the theory of planned behavior and the technology acceptance model. Primary outcome of the study was use of a consumer-grade PA monitor in the past month, analyzed with a stepwise logistic regression model, including psychosocial determinants, technology acceptance and PA and past tracking behavior.

**Results:**

Of the participants, 40 % reported using a PA monitor in the past month. Demographics and psychosocial determinants of PA explained 12 % of PA monitor use. The odds for using a PA monitor was higher with higher feelings of autonomy (1.772; CI:1.128–2.783). Adding technology acceptance to the regression model increased the explained variance to 66 %, with significant ORs of perceived ease of use (2.403; CI:1.479–3.904), perceived usefulness (0.405; CI:0.224–0.731), attitude towards PA monitors (2.235; CI:1.222–4.087), affective quality (2.293 CI:1.252–4.201), intention to use PA monitors in the near future (4.174; CI:2.320–7.512), and subcultural appeal (0.660; CI:0.455–0.958).

**Conclusions:**

This study confirmed the value of integrating consumer-grade PA in clinical trials, since they were used regardless of the amount of leisure time PA, motivation, age, and educational level. This indicates that trials that use people's own PA trackers to recruit and screen participants or in interventions likely includes a generalizable sample. Furthermore, the results provide concrete pointers within technology acceptance that could contribute to recruitment in trials relying on participants' own PA monitors.

## 1 Introduction

To optimize enrollment in clinical trials, researchers are constantly looking for ways to reduce the burden on participants. A great opportunity is to use measurement methods already adopted by the target population, like wearables for physical activity (PA) monitoring [1,2]. Participants' experience can significantly be improved by incorporating PA data from these wearables either as a means to reach and recruit participants [3,4], to evaluate changes in behavior [5,6], or to incorporate in interventions [7,8]. Data can be retrieved from a distance, without multiple visits to the lab, which are typically necessary with most research-grade PA monitors*.* Moreover, the consumer market for PA monitors has rapidly expanded due to increased user-friendliness, falling prices and availability. Incorporating people's own PA monitors enables objective measurement of PA in any clinical trial, since they are user-friendly and already in use. These monitors have enabled nation-wide interventions and monitoring [3,4]; promising proof of concept that PA monitors offer great opportunities as a route for recruitment for clinical trials. Consumer-grade monitors estimate PA levels, where especially steps taken is a promising standardized, universal metric [1]; prevailing examples are accelerometers in smartphones and wrist-worn activity monitors, like FitBit™, RunKeeper™, and Apple watch™. Generally, consumer-grade PA monitors showed acceptable validity, and notably high reliability for assessing step count [9]. The strength of using consumer-grade PA monitors lies in the premise that a significant proportion of target populations have already adopted the technology. Hence, if behavioral interventions can be built around the consumer's PA monitor, the decision to join a study may be more favorable. Allowing participants to use their own (consumer) PA monitor rather than research devices may be advantageous, as it spares them the effort of learning to use unfamiliar technology and incurs no extra costs. Moreover, in all types of trials, data from consumers' monitors can be used to assess at-risk populations or specific eligibility criteria based on PA levels [10] with lower effort than when questionnaires or devices have to be employed, under the condition that a validated measure like step count is used. However, the use of technology, like PA monitors, as evaluation or intervention mode in clinical trials requires efforts to ensure representation and representativeness of a diverse population [11].

Clinical research integrating technology is prone to selection bias, also in PA research [2]. Hence, generalizable conclusions from these trials require knowledge and understanding of the representativeness of its users. Relying on consumer-grade PA monitors imposes the risk of underrepresenting the older, less healthy, less active and less motivated individuals, and those with lower socio-economic backgrounds [[12], [13], [14], [15]], resulting in implications for the representativeness of trial results [2,11]. So far, factors related to the use of PA monitors have been reported predominantly in trials in which PA monitors were supplied by researchers as part of the experimental condition that participants are exposed to [16], or only analyzing (past) users and lacking information about non-users [17]. Insight in factors that differ between users and non-users of monitors in daily life are required to provide recommendations on how consumer-grade PA wearables can be integrated in clinical trials optimally. For example, these insights can result in recommendations on extra efforts to reach certain groups that do not use PA monitors.

Besides demographics that have been discussed previously to impact generalizability (e.g. age and socio-economic status [13,15]), other factors like psychosocial determinants of PA behavior and technology acceptance might differ between the users and non-users of PA monitors. Psychosocial determinants of PA behavior might not only determine whether someone is willing to use a PA monitor, but might also determine the degree in which interventions using a PA monitor are able to influence the PA behavior, and consequently impact the generalizability of effectiveness. To illustrate, the use of PA monitors has previously been related to motivation to increase PA [18]. Consequently, results of an intervention study including participants with their own PA monitor possibly overestimate effectiveness, given the expected higher motivation of this group. Other concepts from behavior change models [19,20], like autonomy, competence, and self-efficacy, determine PA behavior [21] and may be related to the use of PA monitors as well, and hence may be relevant for representative recruitment in clinical trials.

The Technology Acceptance Model (TAM) proposes that perceived usefulness and perceived ease of use of the technology determine the intention to use it [22], and is a key framework to consider in reaching a sizable and generalizable part of population using PA monitors. Perceived usefulness is defined as the degree to which someone believes that using the PA monitor would support their goal, and perceived ease of use refers to the belief that using the monitor will be free of effort [22,23]. Both aspects play a role in the use of PA monitors [23]. However, explained variance of fitness tracker use based on these two TAM aspects alone was under 25 % [24], implying that other aspects related to the functionality of the technology might be overlooked. For example, social norms, subcultural appeal and perceived privacy risk may play a role in deciding to use a PA monitor [[24], [25], [26], [27]]. Insights into relevant aspects of technology acceptance enables future promotion and facilitation of PA monitor adoption by current non-users.

The current study aims to assess which characteristics of the users and non-users of consumer-grade activity monitors are different and comparable with regard to demographics, perceived psychosocial determinants of PA behavior, PA behavior itself, and technology acceptance of PA monitors. The application of the findings of this study is twofold. We aim to indicate which aspects of technology acceptance can aid concrete actions in trial participant recruitment when using consumer-grade activity monitors in clinical trials. Moreover, we aim to identify differences in PA behavior and its psychosocial determinants that imply generalizability issues of trials utilizing these monitors.

## 2 Materials and methods

### 2.1 Design and participants

The study used cross-sectional data collected with an online, web-based questionnaire in LimeSurvey, and was approved by the Research Ethics Committee of the Open University of the Netherlands. Dutch speaking adults of the general populations were invited via social media, email and via personal communication of Open University researchers and students. Participants were 18 years or older and gave informed consent after being informed about the study purpose, data handling and privacy. Other than being over 18 years of age, no inclusion criteria were applied. Informed consent was given on the first page of the online questionnaire. The questionnaire was completed by 534 adults with a mean age of 43 years (±14 years), a BMI of 24 kg/m^2^ (±4 kg/m^2^), and of which 70 % were women. The educational level of 4 % of participants was low (i.e., primary, basic vocational, or lower general school), 24 % was intermediate (i.e., medium vocational school, higher general secondary education, and preparatory academic education), and 73 % was high (i.e., higher vocational school or university level).

### 2.2 Measurements

All participants completed a self-report questionnaire measuring demographic characteristics, PA, psychosocial determinants to PA and technology acceptance regarding PA monitors. Height and weight were used to calculate BMI. To be able to analyze differences between users and non-users, the questionnaire included the question “Did you use a PA monitor in the past 30 days?”. Additionally, participants reported since when they were using PA monitors, and which type of PA monitor was used in the past 30 days: a smartphone, a smart watch, or a device that solely measures PA (and no other functionalities) without connection to other digital devices. Additionally, the main motive for using a PA monitor was assessed bya question based on the definitions by Rooksby et al. [28].

The Short QUestionnaire to Assess Health-enhancing PA (SQUASH) assessed weekly minutes of PA. Previous research has shown acceptable reproducibility and validity of the SQUASH in the general adult population [29]. Due to a technical error PA at school or work could not be included in the calculations. Hence, only minutes of leisure time MVPA per week including exercising, walking, bicycling, gardening were calculated using the SQUASH protocol [29].

Psychosocial determinants of PA from the theory of planned behavior (TPB) [19] and self-determination theory (SDT) [20,21] were measured using existing questionnaires (Table 1). Intrinsic motivation to engage in PA was assessed using the items from the Behavioral Regulation in Exercise Questionnaire (BREQ2) [30]. Intention to be sufficiently physically active was measured with a questionnaire by Sheeran and Orbell [31]. Measures for attitude, self-efficacy and social influence for being sufficiently physically active were included as adapted by Peels et al. [32], based on Resnick et al. [33]. Action planning of PA was measured with a questionnaire by Lippke, Ziegelmann and Schwarzer [34]. Feelings of autonomy, competence and relatedness regarding PA were assessed using the Basic Psychological Needs in Exercise Scale (BPNES) [35]. Although it is not part of the TPB and SDT, competition was added to this study because of the frequent use of competitive elements in PA tracking [36]. The scale for competition was self-constructed.

**Table 1:** Psychosocial determinants of PA included in the study.

Concept	No. of items	Item example	Cronbach's alpha
Intrinsic motivation	4	‘I engage in PA because it is fun’ *1 (totally disagree) – 5 (totally agree)*	0.921
Intention	3	‘Are you planning to be sufficiently physically active? *1 (definitely not) – 10 (yes, definitely)*	0.931
Attitude (pros/cons)	7/6	‘I find being regularly physically active enjoyable/very time consuming’ *1 (totally disagree) – 5 (totally agree)*	0.875/0.688
Self-efficacy	10	‘Do you find yourself able to be physically active for at least 30 min per day when you are tired?’ *1 (definitely unable) – 5 (definitely able)*	0.886
Competition	7	‘I enjoy comparing my PA level to that of others of my age.’ *1 (totally disagree) – 5 (totally agree)*	0.869
Action planning	6	‘I plan when to do my physical activity’ *1 (never) – 5 (always)*	0.910
Social influence	4	‘Is your family sufficiently active?’ *1 (never) – 5 (always)*	0.633
Competence	4	‘I feel I have made a lot of progress in relation to the goal I want to achieve.’ *1 (totally disagree) – 5 (totally agree)*	0.804
Relatedness	4	‘My relationships with the people I exercise with are close.’ *1 (totally disagree) – 5 (totally agree)*	0.876
Autonomy	4	‘The way I exercise is in agreement with my choices and interests.’*1 (totally disagree) – 5 (totally agree)*	0.804

Concepts from the general technology acceptance models including TAM and UTAUT [22,37,38] were complemented with potentially relevant technology acceptance concepts that may play a role in use of wearables in health care and smart watches [26,27] (Table 2). Questionnaires for attitude, perceived ease of use, perceived usefulness, affective quality, relative advantage, mobility, availability, subcultural appeal and costs were applied to PA monitors based on a questionnaire used by Kim and Shin to assess acceptance for smart watches [26]. Questionnaires regarding performance expectancy, hedonic motivation, effort expectancy, social influence, functional congruence, self-efficacy, perceived privacy risk and behavioral intention to use a PA monitor in the future were adapted from Gao et al. [27]. Cronbach's alphas were determined for all scales (Table 1, Table 2). Earlier use was assessed by one item asking whether a PA monitor was ever used prior to the past month.

**Table 2:** Concepts of technology acceptance regarding PA monitors included in the study.

Concept	No. of items	Item example	Cronbach's alpha
Attitude	4	‘Using a PA monitor is a good idea’ *1 (totally disagree) – 5 (totally agree)*	0.938
Perceived ease of use	3	‘I find a PA monitor easy to use’ *1 (totally disagree) – 5 (totally agree)*	0.898
Perceived usefulness	5	‘A PA monitor is useful in reaching my PA goals’ *1 (totally disagree) – 5 (totally agree)*	0.947
Affective quality	3	‘A PA monitor is attractive and pleasing’ *1 (totally disagree) – 5 (totally agree)*	0.820
Relative advantage	3	‘The advantages of using a PA monitor outweigh the disadvantages’*1 (totally disagree) – 5 (totally agree)*	0.854
Subcultural appeal	5	‘If I use a PA monitor, it would make me stand apart from others’ *1 (totally disagree) – 5 (totally agree)*	0.926
Costs	3	‘A PA monitor is expensive’ *1 (totally disagree) – 5 (totally agree)*	0.642
Performance expectancy	3	‘I find a PA monitor useful in my daily life’ *1 (totally disagree) – 5 (totally agree)*	0.884
Hedonic motivation	3	‘Using a PA monitor is fun’ *1 (totally disagree) – 5 (totally agree)*	0.925
Effort expectancy	2	‘It is easy for me to become skillful at using a PA monitor’ *1 (totally disagree) – 5 (totally agree)*	0.947
Social influence	3	‘People who are important to me would think that I should use a PA monitor’ *1 (totally disagree) – 5 (totally agree)*	0.971
Perceived privacy risk	3	‘It would be risky to disclose my personal health information to vendors providing PA monitors’ *1 (totally disagree) – 5 (totally agree)*	0.937
Functional congruence	3	‘I expect a PA monitor to be comfortable’ *1 (totally disagree) – 5 (totally agree)*	0.705
Self-efficacy	3	‘It is easy for me to self-monitor my physical condition by using a PA monitor’ *1 (totally disagree) – 5 (totally agree)*	0.876
Availability	4	‘A PA monitor offers the sense of real-time connectedness’ *1 (totally disagree) – 5 (totally agree)*	0.881
Mobility	3	‘I feel I can use a PA monitor anywhere’ *1 (totally disagree) – 5 (totally agree)*	0.806
Intention	3	‘I intend to use a PA monitor in the future’ *1 (totally disagree) – 5 (totally agree)*	0.925

### 2.3 Analyses

First, all independent variables were standardized. A hierarchical logistic regression analysis was used to identify concepts related to using a PA monitor in the past month. The predictors were added to the logistic model in four blocks, the first block contained the distal concepts and the final block the most proximal concepts: 1) demographic characteristics (i.e. age, sex (male/female), educational level and BMI); 2) psychosocial determinants of PA; 3) technology acceptance concepts regarding PA monitors; and 4) minutes of leisure time MVPA and earlier use of a PA monitor. The analyses were checked for multicollinearity with the variance inflation factor (VIF). The odds ratios (*OR*s) and the 95 % confidence intervals are presented. Explained variance of the logistic models was estimated with Nagelkerke *R*^*2*^ [39]. The measures of this study were all self-reported questionnaires, potentially causing common method bias [40]. Harman's single factor test was used to assess common method bias in the independent variables in the regression model. Analyses were performed in SPSS version 24 with a significance level of 5 %.

## 3 Results

### 3.1 Participant characteristics

Of the participants, 40 % (N = 215) reported using a PA monitor or app in the past month. In users, the main motives reported for using a PA monitor were documentary tracking (65 %), directive and diagnostic tracking (13 % each), collecting rewards (5 %), and fetishised tracking (3 %) [28]. Of the 534 participants, 65 were excluded from the analysis due to missing data; main participant characteristics of the excluded participants (i.e. sex, age, BMI, educational level, use of PA monitor and PA level) did not differ significantly from the participants included in the analysis. The majority of participants reported to have used one type of PA monitor in the past month (n = 188; 87.4 %), whereas 10.7 % and 1.9 % reported to have used respectively two and three types of PA monitors. Using a smartwatch that tracks PA was reported by 109 participants (50.9 %), an app on the phone by 91 participants (42.3 %), a PA monitor unconnected to phone, smartwatch, tablet or PC by 37 participants (17.2 %). Six participants (2.8 %) reported using a device which did not fit these three categories, and did not report which device they had been using. The average reported time of use was 32 months (±36 months).

### 3.2 Demographics characteristics

For all models, all independent variables showed a VIF value of below 5. The Harman's single factor test revealed a percentage of 24.5, indicating no presence of common method bias, warranting no further actions in the statistical analyses. The regression model comprising only demographics explained 5 % of the variance and showed that lower age was associated with a higher probability of PA monitor use (*OR* = 0.703, CI 0.577–0.856, *p* < 0.001; Table 3). Sex, educational level and BMI were not related to PA monitor use. Adding the psychosocial determinants of PA to the model did not significantly influence the impact of the demographics on PA monitor use. When concepts of technology acceptance were added in the third block of the regression model, age was no longer associated with use of a PA monitor (*p* = 0.532).

**Table 3:** Results of the hierarchical logistic regression analysis (N = 469).

	Model 1 *R*^2^ = 0.049	Model 2 *R*^2^ = 0.115	Model 3 *R*^2^ = 0.660	Model 4 *R*^2^ = 0.665
	*OR* (CI)	*OR* (CI)	*OR* (CI)	*OR* (CI)
*Step 1: Demographic characteristics*				
Sex (female = 0)	1.186 (0.781–1.802)	1.209 (0.783–1.867)	1.018 (0.528–1.960)	0.965 (0.524–1.966)
Age	0.703 (0.577–0.856)∗	0.714 (0.576–0.885)∗	0.900 (0.646–1.253)	0.874 (0.624–1.225)
Medium education (low = 0)	0.683 (0.244–1.912)	0.619 (0.210–1.825)	0.400 (0.069–2.299)	0.439 (0.075–2.553)
High education (low = 0)	1.098 (0.419–2.881)	1.059 (0.383–2.926)	0.839 (0.158–4.446)	0.991 (0.184–5.346)
BMI	1.094 (0.902–1.326)	1.181 (0.953–1.463)	0.983 (0.705–1.372)	0.996 (0.709–1.397)

*Step 2: Psychosocial determinants of PA*				

Intrinsic motivation		0.886 (0.634–1.240)	0.773 (0.471–1.267)	0.794 (0.483–1.303)
Intention		1.286 (0.942–1.756)	1.165 (0.739–1.835)	1.140 (0.721–1.803)
Attitude (pros)		1.225 (0.871–1.721)	1.008 (0.608–1.672)	0.972 (0.585–1.613)
Attitude (cons)		1.071 (0.838–1.367)	1.047 (0.709–1.548)	1.024 (0.687–1.527)
Self-efficacy		0.883 (0.672–1.161)	0.989 (0.656–1.491)	0.934 (0.606–1.437)
Competition		1.055 (0.854–1.302)	1.175 (0.839–1.644)	1.143 (0.814–1.603)
Action planning		1.391 (1.104–1.763)∗	1.382 (0.940–2.032)	1.390 (0.936–2.064)
Social influence		0.941 (0.763–1.159)	0.992 (0.730–1.347)	0.963 (0.710–1.307)
Competence		1.064 (0.782–1.448)	0.900 (0.574–1.411)	0.881 (0.560–1.384)
Relatedness		0.970 (0.750–1.254)	0.680 (0.456–1.013)	0.671 (0.448–1.006)
Autonomy		1.019 (0.766–1.356)	1.755 (1.118–2.754)∗	1.772 (1.128–2.783)∗

*Step 3: Technology acceptance concepts regarding PA monitors*				

Attitude			2.203 (1.213–4.001)∗	2.235 (1.222–4.087)∗
Perceived ease of use			2.288 (1.425–3.673)∗	2.403 (1.479–3.904)∗
Perceived usefulness			0.409 (0.228–0.736)∗	0.405 (0.224–0.731)∗
Affective quality			2.325 (1.276–4.235)∗	2.293 (1.252–4.201)∗
Relative advantage			0.904 (0.519–1.576)	0.916 (0.521–1.610)
Subcultural appeal			0.661 (0.459–0.954)∗	0.660 (0.455–0.958)∗
Costs			0.850 (0.609–1.187)	0.842 (0.601–1.178)
Performance expectancy			0.832 (0.488–1.417)	0.805 (0.469–1.379)
Hedonic motivation			1.352 (0.767–2.384)	1.372 (0.771–2.443)
Effort expectancy			0.795 (0.505–1.252)	0.805 (0.508–1.274)
Social influence			0.856 (0.606–1.209)	0.853 (0.601–1.211)
Perceived privacy risk			1.559 (1.130–2.150)∗	1.572 (1.136–2.176)∗
Functional congruence			1.584 (1.065–2.356)∗	1.645 (1.102–2.455)∗
Self-efficacy			0.779 (0.489–1.242)	0.744 (0.463–1.196)
Availability			0.821 (0.540–1.247)	0.834 (0.548–1.271)
Mobility			1.237 (0.693–2.208)	1.293 (0.719–2.325)
Intention for PA monitor			4.028 (2.262–7.172)∗	4.174 (2.320–7.512)∗

*Step 4: PA level and previous use*				

Prior use of PA monitor				0.613 (0.313–1.203)
Leisure time MVPA				1.277 (0.906–1.800)

*OR* = Odds ratio; *CI* = Confidence intervals, *R*^*2*^ = Nagelkerke R squared; ∗*p* < 0.05. Means and standard deviations of each independent variable are summarized in appendix 1.

### 3.3 Psychosocial determinants of physical activity

Adding psychosocial determinants of PA as the second block (Table 3) improved the model significantly (*p* = 0.012), explaining 12 % of the variance. Higher level of action planning was related to a higher probability to use a PA monitor (*OR* = 1.391, CI 1.104–1.763, *p* = 0.005). When concepts of technology acceptance were added as the third block, the relationship of action planning with PA monitor use was no longer significant (*OR* = 1.382, CI 0.940–2.032, *p* = 0.100). Increased feelings of autonomy however, did become related to PA monitor use (*OR* = 1.755, CI 1.118–2.754; *p* = 0.014). All other concepts showed no associations.

### 3.4 Technology acceptance concepts regarding physical activity monitors

Addition of the acceptance concepts regarding PA monitors increased the explained variance significantly to 66 % (*p* < 0.001). A more positive attitude towards PA monitors (*OR* = 2.203, CI 1.213–4.001, *p* = 0.009), higher levels of perceived ease of use (*OR* = 2.288, CI 1.425–3.673, *p* = 0.001), affective quality (*OR* = 2.325, CI 1.276–4.235, *p* = 0.006), perceived privacy risk (*OR* = 1.559, CI 1.130–2.150, *p* = 0.007), functional congruence (*OR* = 1.584, CI 1.065–2.356, *p* = 0.023), and intention to use a PA monitor (*OR* = 4.028, CI 2.262–7.172, *p* < 0.001) were associated with higher odds for using a PA monitor. In contrast, levels of perceived usefulness (*OR* = 0.409, CI 0.228–0.736, *p* = 0.003) and subcultural appeal (*OR* = 0.661, CI 0.459–0.954, *p* = 0.027) were negatively associated with using a PA monitor.

### 3.5 PA level and previous use

Prior use of a PA monitor and leisure time MVPA (added as the final block) did not improve the model (explained variance of 67 %, *p* = 0.158) and had no relationship with current PA monitor use.

## 4 Discussion

The current study aimed to compare the characteristics of users and non-users of consumer-grade PA monitors in terms of demographic characteristics, psychosocial determinants of PA behavior and technology acceptance regarding PA monitors. With the results, researchers conducting clinical trials with PA consumer-grade PA monitors can be informed about the expected representativeness of the sample and ways to increase recruitment rate. The hierarchical analyses revealed that all three categories contributed to the likelihood for using a PA monitor. Demographic characteristics and psychosocial determinants of PA behavior explained only 12 % of the variance, and of those categories only a higher feeling of autonomy regarding PA behavior was related to using a PA monitor. Adding technology acceptance to the model had substantial impact. The contribution of technology acceptance to the model and the moderate to strong associations between many technology acceptance concepts and PA monitor use, indicate that perceptions of PA monitors play an important role.

Our findings indicate that the role of psychosocial determinants of PA behavior in using PA monitors is present, but not as imperative as the role of technology acceptance. The poor explained variance of those psychosocial determinants is reflected in the insignificant *OR*s, with the exception of autonomy regarding PA behavior. Participants feeling more in control over one's PA behavior were more likely to use a PA monitor. Given the cross-sectional design of the study, this might either mean that people with higher feelings of autonomy are more inclined to adopt a PA monitor, or that PA monitors are able to increase autonomy. The latter is promising for health promotion, especially when combined with effective behavior change techniques in interventions [41,42]. Other psychosocial determinants were not associated with PA monitor use, so one may conclude that PA monitor use is not actually related to peoples' beliefs about PA. However, peoples attitude, motivation, self-efficacy to be physically active, might already be translated into higher technology acceptance of PA monitors.

Over 50 % of the variance of using a PA monitor was explained by technology acceptance, providing concrete pointers to promote the use of PA monitors. Building upon a previous study showing that the concepts from TAM predicted 25 % of fitness monitor use [24], our study confirms that technology acceptance plays a substantial role in the use of PA monitors. The key concepts of TAM, i.e. perceived ease of use and usefulness, were related to the odds for using a PA monitor, as were other technology acceptance concepts. Remarkable are the negative associations of perceived usefulness and subcultural appeal with PA monitor use in the current study. Perceived usefulness in our study was formulated as the usefulness of a PA monitor to reach PA goals, assuming goal driven tracking as motive of PA monitor use. However, only 13 % of the users indicated directive monitoring (i.e. goal driven tracking) as their motive for using a PA monitor, whereas the majority of the users in our study (65 %) indicated documentary tracking as their motive, i.e. gaining insight in current activities without the goal to change [28]. A high prevalence of documentary tracking might have resulted in lower levels of perceived usefulness since the questionnaire items were aimed at reaching goals. The other surprising finding was that subcultural appeal was negatively related to PA monitor use. In previous studies, higher subcultural appeal led to a more positive attitude towards smart watches as part of the ‘coolness’ of being distinguished from others who have not adopted this innovation [26,43]. Yet, the current state of adoption of PA monitors raises the question whether using this technology is still unique enough for people to feel part a subculture. In line with this, we did not find a significant role of age in our study, whereas users of PA monitors were generally younger in previous studies [13,44,45]. Looking at the Diffusion of Innovations theory [46], one might argue that the subcultural appeal diminishes when the majority has adopted an innovation. At that point of diffusion, using a PA monitor will not make you stand out in a crowd. Jarrahi et al. also argue that for non-motivated adopters the appeal and novelty of Fitbit devices decrease over time [18]. Hence, the influence of subcultural appeal on adoption is probably not constant and might be larger when the overall adoption rate is lower. Additionally, it should be noted that age was significant in the first models, but this relationship disappears after adding concepts of technology acceptance in the model. This indicates a potential mediation pathway, in which older participants have lower technology acceptance levels and hence lower odds to use a PA monitor. The insight into technology acceptance is highly relevant, since studies using person-generated health data like PA, for either eligibility, interventions and/or evaluation, rely heavily on devices owned by the participant [47].

High intention for future use was strongly associated with the probability of current use which is in line with research among older adults [25]. In agreement with other studies, attitude, perceived ease of use and affective quality played their part in explaining (intended) use of PA monitors and smart watches [23,24,26]. In addition, perceived privacy risk, and functional congruence were related to PA monitor use.

In our study, PA level did not predict the use of PA monitors, in contrast to earlier findings [45,48,49]. However, studies that did find a relationship, compared inactive and active people [45,49] or compared inactive, insufficiently active and active people [48], hindering solid comparison with our study results. An important note to make here, is that we studied leisure time MVPA, excluding PA during work and/or school, and similar to previous studies [45,48,49] we relied on self-report data. Regardless of this, we expected that PA monitor users had a higher PA level than non-users. However, our results indicate that voluntarily adopted PA monitors reach people regardless of PA level during leisure time, which is promising since the risk of increasing health inequalities thus might be lower than expected.

One of the limitations of the current study was the cross-sectional design which refrained us from assessing causal relationships. This limited any conclusions about mediation processes that might be suggested by the findings that the *OR*s of action planning and age were no longer significant when technology acceptance was added to the model. Technology acceptance and intention are likely to mediate the relationship between age and PA monitor use. Based on our results, a relationship between age and PA monitor use cannot be ruled out. The advanced UTAUT-model by Venkatesh and colleagues [37] and the Theory of Planned Behavior hypothesize that actual behavior is predicted by intention to act (e.g. use a PA monitor), which is in turn influenced by the distal determinants. More insight in the causality of this relation in the general population is needed as they provide implications for the reach of PA monitors. Additionally, although our statistical test indicates no presence of common method bias, it needs to be acknowledged that Harman's single factor test cannot rule out the occurrence of common method bias [50]. Prevention of systematic effects of common method bias should be an integral part of designing future research [40,51]. In our study some factors may have caused common method bias: self-administration, the length of the questionnaire, the frequent use of a Likert-scale, and asking people not using PA monitors about their opinion of the monitors. Based on the questionnaire, we cannot be sure whether participants actually did or did not use a PA monitor, and how consistent and engaged the use was between participants. Hence, there can be participants in the ‘yes’-group who abandoned the use of the PA monitor after one attempt, potentially leading to underestimations of the differences between frequent users and non-users. To enhance the validity of the item to assess use of the PA monitor, we additionally asked for the type of monitor in use, and since when they are using PA monitors. However, in future studies, we advise to take variability of usage into account and measure use objectively. Another weakness of this study is the relatively large proportion of women and low proportion of low educated participants, which limits generalizability of the results. Specifically, the relatively high proportion of highly educated participants and the use of an online questionnaire increases the risk of excluding those with lower technology literacy.

A strength of our study was the inclusion of both users and non-users, which is crucial to guide further research using PA monitors and to stimulate PA monitor adoption and participant recruitment. Earlier studies mainly involved users only, of whom the characteristics are very important to know for further development of PA monitors. Nonetheless, a large proportion of the population who should be reached with PA interventions currently do not use PA monitors. Another strength is the real-life setting, as we focused on voluntary PA monitor use in daily life, which is distinctively different than using a wearable provided by researchers, and is especially important for future implementation in clinical trials.

## 5 Conclusion

The current study confirmed that consumer-grade PA monitors offer a valuable opportunity to integrate in clinical trials, since they were used regardless of the amount of leisure time PA, level of motivation, age, educational level, and other relevant aspects. This indicates that when trials use people's own PA trackers to recruit and screen participants or use it in interventions, even people who are less active, less motivated, and older can be reached, enhancing the representativeness of findings. Since autonomy for PA was significantly higher in users of PA monitors, it is recommended to monitor this determinant while evaluating interventions that incorporate PA monitors or rely on current users. Moreover, when attempting to recruit participants for trials including PA monitors, several concepts of technology acceptance seem important to stimulate enrollment. Specifically perceived ease of use, affective quality and general attitude towards PA monitors showed high *OR*s, indicating that recruitment may benefit from both ensuring and highlighting the personal advantages and ease of use of the monitors. Future studies should give special attention to the role of perceived usefulness and motives for using PA monitors.

## CRediT authorship contribution statement

**Brenda A.J. Berendsen:** Writing – original draft, Supervision, Methodology, Investigation, Formal analysis, Conceptualization. **Rianne H.J. Golsteijn:** Writing – review & editing, Data curation. **Lilian Lechner:** Writing – review & editing, Supervision. **Catherine Bolman:** Writing – review & editing. **Denise A. Peels:** Writing – review & editing, Supervision, Methodology, Investigation, Conceptualization.

## Consent to participate and publish

Informed consent was obtained from all individual participants included in the study.

## Ethics approval

This observational study was reviewed and approved by the Committee for Ethics and Consent in Research of the Open University of the Netherlands (reference number U2018/04219/NGU).

## Data availability

The data that support the findings of this study are available from the corresponding author, upon reasonable request.

## Funding

This research did not receive any specific grant from funding agencies in the public, commercial, or not-for-profit sectors.

## Declaration of competing interest

The authors declare that they have no known competing financial interests or personal relationships that could have appeared to influence the work reported in this paper.

## Figures and Tables

**Table:** 

	Mean	Standard Deviation
*Demographics*		
Age (years)	43	14
BMI (kg/m^2^)	24	4
*Psychosocial determinants*		
Intrinsic Motivation	4.0	0.8
Intention (1-10)	4.1	1.0
Attitude pros	4.1	0.6
Attitude cons	2.0	0.6
Self-Efficacy	3.6	0.7
Competition	2.2	0.8
Action Plans	3.1	0.9
Social Influence	3.4	0.7
Competence	3.5	0.8
Relatedness	3.6	0.8
Autonomy	3.7	0.8
*Technology acceptance*		
Attitude	3.6	0.9
Perceived Ease of Use	3.8	0.8
Perceived Usefulness	3.3	0.9
Affective Quality	3.2	0.8
Relative Advantage	3.4	0.8
Subcultural appeal	2.1	0.8
Costs	2.7	0.7
Performance Expectancy	3.0	0.9
Hedonic Motivation	3.3	0.9
Effort expectancy	3.9	0.8
Social Influence	2.0	0.8
Perceived Privacy Risk	3.0	0.9
Functional Congruence	3.6	0.7
Self-Efficacy	3.6	0.8
Availability	3.4	0.8
Mobility	3.7	0.8
Behavioral Intention	2.9	1.1
*Physical activity*		
PA level (min/week)	744.5	588.1
